# Factors influencing performance and injury risk in elite female Gaelic team sport players and future research directions: a narrative review

**DOI:** 10.1186/s13102-022-00553-8

**Published:** 2022-09-02

**Authors:** John David Duggan, Kieran Collins, Karen Keane

**Affiliations:** 1 Department of Sport, Exercise & Nutrition, School of Science & Computing, Atlantic Technological University Galway, Dublin Road, Galway, Ireland; 2 Gaelic Sports Research Centre, Technological University Dublin-Tallaght Campus, Tallaght, Dublin 24, Dublin, Ireland

**Keywords:** Elite female Gaelic sports, Gaelic games, Performance, Nutrition, Injury, Anthropometrics

## Abstract

**Background:**

Sports science research in elite female Gaelic team sports has increased in recent years, but still a large disparity exists between the volume of studies involving male and female players. As a consequence of this, it is difficult for practitioners to develop an evidence-based approach when working with female players.

**Main body:**

In this review, we discuss the current research available in elite female Gaelic team sports with focus on seven specific areas including physical and physiological demands, anthropometric and performance characteristics, injury risk, nutritional considerations, and female physiology. There appears to be unique physical demands data in match play across positions in Camogie, however, there is currently no comparative data available in ladies Gaelic football. Similarly, there is no research available on the physiological demands of both elite female Gaelic team sports. According to existing literature, performance characteristics such as speed and power are lower in this population compared to other elite female team sports. Although data is limited, the anthropometric characteristics of elite female Gaelic team sport players appear homogenous with some positional differences observed at a sub-elite level. Previous research has demonstrated a high prevalence of lower limb injuries in female elite Gaelic team sports and the provision of quality, evidence-based strength & conditioning could help mitigate these injury risks. Female Gaelic team sport players have been shown to have poor nutrition knowledge and inadequate intakes of micronutrients. Finally, although menstrual cycle phase and oral contraceptives have been shown to influence performance in other female intermittent sports, to date there has not been any research carried out in elite female Gaelic team sport players.

**Conclusions:**

It is evident that limited research has been carried out on elite female Gaelic sport players. More up-to-date, high-quality investigations are needed to address the research gaps, which in turn should enable practitioners in the field to apply sound, evidence-based practice/theory when working with this population.

## Background/introduction

Camogie [kuh mow gee] and ladies Gaelic football are two of the most popular female participation-based sports in Ireland. Furthermore, as the Irish diaspora settle around the world and the increasing globalization of sport, both games are now played in every corner of the globe. Despite the amateur ethos of Gaelic games, inter-county (elite) female Gaelic team sport players complete up to six tactical, technical and physical training sessions per week, consisting of combination of both pitch and resistance training-based sessions [1, 2]. During the competitive season, elite inter-county female Gaelic team sport players compete in two major competitions, the National League, and the All-Ireland Championship. The National League runs from January to April, whilst the All-Ireland Championship runs from May to July each year. From a competition/training perspective, during the peak competitive phases of the National League and All-Ireland, female athletes may partake in games on a weekly/fortnightly basis depending on progress through each of the major competitions.

In both female Gaelic team sports, the game model of both sports is similar [3]. A team consists of 15 players, with the possibility of using five substitutes [4]. Each team includes a goalkeeper, two lines of three defensive players (full-back and half-back), two midfielders, and two lines of attacking players (half forward and full forward). Games at elite inter-county level comprise of two 30-min halves played on a rectangular pitch, 145 m in length and 90 m in width [5]. The principles of play of both female Gaelic team sports are to disrupt and unbalance the defense by striking the Gaelic football or the solid leather slíotar [slit er] (camogie) through the oppositions goalposts (similar to rugby goalposts), either below the cross bar for a goal (three points) or above for a point [1]. In ladies Gaelic football (similar principles of play to Australian Rules Football and basketball), the ball (diameter 680 mm, mass 480 g) is spherical, and heavier than the ball used in soccer. The skills of the ladies Gaelic football include high catching/fielding, handling the ball, kicking the football over a range of distances, solo running with the ball, passing the ball by hand, and blocking and intercepting [4]. Successful ladies Gaelic football teams were observed to have significantly superior ability to gain and use possession [6]. In camogie, (similar principles of play to field hockey & Lacrosse), an ash stick (hurley) is used to strike the slíotar (diameter 69–72 mm, mass 110–120 g) over variable distances. The skills of camogie include high catching/fielding, aerial duels, striking the sliotar over short and long distances, passing the slíotar by hand, solo running with the slíotar balanced on the hurley, blocking opponents using the hurley and intercepting the slíotar with the hurley or by hand [1, 4].

There has been an increase in both participation and professionalization of female team sports in the past decade [6, 7]. Despite this, there appears to be an underrepresentation of sports science research focused on female athletes [8, 9]. It has been reported that only 4% of research conducted in sport science and medicine includes female-only cohorts [10, 11]. This disparity influences practitioners to apply evidence developed in male team athletes’ environments to the female team sport domain, which has the potential to lead to erroneous assumptions [9, 12]. Progressively, there has been recent research efforts to compile evidence on the match demands, physiological characteristics and health and performance considerations of female athletes including soccer [13, 14], rugby 7 s [15] and AFL [16]. However, no such source exists for female Gaelic team sports. Therefore, the primary aim of this review is to summarize existing literature relating to elite female Gaelic team sports athletes including physical and physiological demands, anthropometric and performance characteristics, injury risk, nutritional considerations, and female physiology. In doing so, the authors aim to highlight what is currently known on these specific topics and identify gaps in the research. The review will focus on research conducted in elite female and sub elite Gaelic team sports athletes with comparisons made to other female team sports and male Gaelic team sport athletes when appropriate.

## Physical demands

The use of microtechnology devices such as global positioning systems (GPS) has become ubiquitous in team sports settings to enable sports practitioners to collect comprehensive and real-time data during training and competition [17]. The GPS data can aid practitioners to objectively quantify the physical demands of competition, examine individual positional workloads and establish training intensities [18]. Despite the growing number of GPS research in the male versions of Gaelic team sports [19–23], research in elite female Gaelic team sports is still at the emergent stage.

Currently, just four peer reviewed studies have been conducted on the physical demands of elite female camogie players. The first study demonstrated that in 60 min of camogie match-play, elite players covered 5881 ± 906 m in total, 546 ± 259 m in high-speed running, and 183 ± 130 m sprinting [24]. A second study reported that players covered 5800 ± 900 m in total distance, 733 ± 245 m in high-speed running and 206 ± 93 m in sprinting [25]. Positional and running temporal demands measured during 5 min time epochs in elite camogie suggested that midfielders had the greater total and sprint distance decrements and defenders also exhibited total and relative decrements in match-play [26]. However, caution is warranted using this approach, as it may underprepare athletes for competition and not be representative of the complex demands of match play [27]. This data is comparable to other female team sports including elite female AFL where players, across the range of positions covered 6.0–7.0 km at 95.0–126.0 m min^−1^ during 80 min of match-play [16]. However, the very-high speed running (> 18 km h^−1^) and sprint distances (> 20 km h^−1^) were marginally less for these AFL players when compared to camogie, ~ 417 m and ~ 159 m respectively. Furthermore, it has been shown in camogie that full backs cover less total distance (m) and relative distance (m min^−1^) compared to other positions, whilst half backs and half forwards had higher sprint distances when compared to other playing positions [24]. This is similar to data in elite female field hockey where forwards were reported to have the highest peak output for relative high-speed distance (119.3 ± 19.7 m min^−1^) in comparison to the defenders (100.7 ± 19.7 m min^−1^) [28]. The maximal velocity reported in elite camogie players has been previously reported as 24.9 ± 1.6 km h^−1^ [24], 24.7 ± 1.26 km h^−1^ [20] and most recently 25.4 ± 1.5 km h^−1^ [29], respectively. These values are slightly higher in comparison to elite Irish female rugby players where maximal velocity has been reported as 23.4 ± 2.52 km h^−1^ [30].

Currently, there appears to be no peer-reviewed GPS data for elite female Gaelic football. An early study using time motion analysis investigated player locomotion in elite female Gaelic football and concluded that players spend more time standing still with less time in high intensity activities in comparison to males, with no performance decrements observed across halves of play [31]. However, this method has since been described as outdated. As a result of the paucity of data in ladies Gaelic football, practitioners and coaches rely heavily on data from other female team sports and from the male game, which has received criticism [32]. The use of absolute thresholds is a common example of this, as values from men's Gaelic football have been adopted and applied in the female game [24]. It has previously been suggested that employing male-related speed velocity zones thresholds to the female team sports context could result in an underestimation of workloads [33, 34], and as such female specific high-speed running velocity thresholds have been recommended due to the physiological sex differences in physical fitness/capacity [29, 33–37]. Therefore, an up-to-date analysis of physical demands of elite female Gaelic football using GPS is needed. This information would aid practitioners to design sport and position specific training programs which mimic the unique demands of the game.

## Physiological demands

The use of heart rate (HR) measurement has been suggested previously as an indirect measure of exercise intensity and has become a widespread practice in female team sports [13, 38]. However, despite this there has been no research on HR indices in either elite female Gaelic team sports. The application of HR measures during training and match-play within female Gaelic team sports would ensure that athletes receive an adequate internal training load stimulus and can provide useful feedback in maintaining or improving fitness capacities in preparing for the demands of match-play [32]. HR monitoring could also enable practitioners to design training units to potentially replicate the demands of the game and provide an individualized aerobic stimulus [39, 40]. In elite female soccer, small-sided games elicited a higher HR response (> 85 HR_max_) when compared to medium- and large-conditioned games [39]. This information could be used by Gaelic game practitioners to manipulate the constraints within a modified game to elicit a specific physiological adaptation whilst continually developing the technical and tactical elements of the game [41]. It has previously been shown in elite female soccer players the average HR during competitive games ranged between 152 and 186 b min^−1^, the equivalent ~ 80 and 90% of HR_peak_ [35, 38], with no differences across positions. In contrast, during competitive match play in elite female hockey players, HR_peak_ was reported at 199 ± 1 b min^−1^, with an average HR intensity of 86 ± 7.8 of HR_peak_. Positional differences were also reported with defenders spending more time at > 85% HR_peak_ when compared to forwards (*p* ≤ 0.001) [39]. There is a dearth of the research available on the physiological demands in elite female Gaelic team sports. Further research is needed to examine if HR values are similar and/or if positional differences exist in female Gaelic team sports.

A well-developed aerobic system is required for team sport athletes to adequately recover between periods of play and between periods of maximal and submaximal work [42]. Limited data exists on the performance profile of elite female Gaelic team sports athletes. The estimated aerobic power values for successful elite female Gaelic footballers tend to be high, supporting the belief that there is a large aerobic contribution to playing the game. Keane et al. [43] reported a mean value of 49.9 ± 4.2 mL kg^−1^ min^−1^ for elite female Gaelic footballers. The values compare favorably to elite female soccer (51.9 ± 5.1 mL kg^−1^ min^−1^) and female Australian football (50.4 ± 6.9 mL kg^−1^ min^−1^) players [13, 44]. Tucker and Reilly [45] reported that the mean of female sub-elite Gaelic football players were slightly lower at 42.0 ± 6.8 mL kg^−1^ min^−1^. At present, there is no comparative data for elite camogie players.

## Performance characteristics

In addition to a well-developed aerobic system, strength, speed and power are all essential bio-motor qualities of female Gaelic team sports [46]. The development of muscular strength and power is an important attribute in elite female Gaelic games athletes to enable them to tolerate the physical demands of the games [43]. Recently isometric strength, using an isometric mid-thigh pull (IMTP) test reported the average peak force was 1938.46 ± 300.17 N and average relative force as 28.72 ± 3.11 N/kg in elite camogie athletes [47]. This is in comparison to IMTP results in elite female rugby union in which the backs and forwards average peak force as 2560.8 N and 2729.8 N respectively [48]. The average relative force was 36.4 N/kg for the forwards and 31.8 N/kg for backs. This could be due to the superior training age of the elite female rugby union athletes and the unique physical characteristics of the game. There is a myriad of research to suggest the importance of resistance training for female athletes to enhance performance and mitigate injury risks [49–55]. Furthermore, enhanced muscular strength for athletic performance has been advocated by Suchomel et al. [49, 50] who suggests that it correlated with rate of force development (RFD), mechanical power output, and sports specific movements (running, jumping, striking) as well as enhanced ability to perform on-field sports specific skills.

Strength training programs for female Gaelic team sports athletes, which includes weightlifting, ballistic, complex training and plyometric movements enhances neural drive, neural activation rates and inter-muscular coordination [1]. It was demonstrated that females who engaged in a 20-week, multi-joint, strength-based training improved strength (neural adaptations, (*p* < 0.05) with no change in muscle cross sectional area [56]. Advanced training methods such as complex training have been shown to improve athletic capabilities in female athletes with a high training age [57]. From a training specificity perspective, complex training has been demonstrated to improve running economy in division 1 female soccer player [57]. Further research is needed to ascertain the efficacy of specific strength training modalities in elite female Gaelic team sports athletes.

### Speed characteristics

Rapid acceleration and sprinting ability are important performance characteristics which allow Gaelic team sports players to reach the ball before the opposition; hence, sprint tests are routinely administered to evaluate performance [58, 59]. In elite camogie, match play sprint and high-speed running distances have been reported as 546 ± 259 m and 183 ± 130 m respectively [24]. O’Grady et al. [29] investigated sprint performance in elite camogie and reported that total sprint distance was 162 ± 102 m with the number of sprints accumulated were 9 ± 5 *n*. The average sprint duration was 3 ± 1 s during competitive, elite match-play. There was also greater number of sprints performed < 20 m in elite, inter-county match-play (7 ± 3 *n*) compared to > 20 m (3 ± 2 *n*) [29]. The mean length of sprints during elite match-play was reported as 17 ± 4 m. The sprints accumulated between 80%-90% of relative velocity was reported as 6 ± 3 *n* and > 90% 3 ± 3 *n* during in elite match-play [29]. From a positional perspective, midfielders and half-forwards had the greatest total sprint distance (208 ± 77 & 200 ± 143 m) and peak speed (26.0 ± 1.5 & 25.7 ± 1.9 km h^−1^) in elite camogie match-play [29]. Tucker and Reilly [45] reported mean 30 m sprint times among elite female Gaelic footballers (5.2 ± 0.2 s). Values for elite camogie players with respect of the shorter distances of 5 (1.20 ± 0.08 s), 10 (2.02 ± 0.08 s) and 20 m (3.45 ± 0.11 s) have recently been reported in the literature [48]. This is in comparison to elite Irish female soccer players where sprint times were reported as 10 m (1.89 ± 0.08 s), 20 m (3.26 ± 0.17 s) and 30 m (4.57 ± 0.26) respectively [60].

The development of both acceleration and sprinting mechanics can develop stride length and decreasing ground contact time, whilst optimizing ground reactive forces and decrease injury rates [61]. Faster athletes can produce a greater vertical impulse in a shorter ground contact time [62]. A further goal of maximal velocity sprinting is to achieve a high stride frequency combined with an optimal stride length [61, 62]. Furthermore, to mitigate against hamstring injuries during the terminal swing phase of sprinting, practitioners within elite female Gaelic team sports athletes are recommended to focus on multi-factorial approach which includes slow, high load eccentric contractions at the knee joint with development of efficient maximal velocity sprinting mechanics using front-side mechanics drilling constraints [63–66]. It is important that elite female Gaelic team sports athletes are exposed to adequate maximal velocity sprinting (> 90% of their maximal velocity) throughout the season which may provide a potential aid against hamstring injuries [65, 66]. Therefore, improving acceleration and maximal velocity mechanics could improve performance as well as reduce the risk of posterior-chain injuries across elite female Gaelic team sports athletes. Further research is needed to determine the effectiveness of acceleration and maximal velocity interventions in elite female Gaelic team sport athletes.

### Power

Muscular power is an important attribute in determining athletic ability and predicting success in sport [4]. The countermovement jump (CMJ) is a widely used criterion and is an appropriate measure of muscular power in Gaelic team sport athletes [45]. Camogie players tend to have lower levels of jump performance (27.5 ± 3.1 cm) when compared to female Gaelic footballers despite having similar masses [43, 47]. In addition, the average reactive strength index (RSI) in elite intercounty Camogie players was reported as 1.18 ± 0.21 [47]. In comparison to collegiate female Gaelic footballers in which the average RSI was reported as 1.22 ± 0.47 [67]. Additionally, in elite Irish female soccer players the average RSI was reported as 1.56 ± 0.25 [60]. Therefore, the implementation of reactive strength-based modalities in elite female Gaelic team sports athletes may lead to improvements in stretch shortening cycle ability. The CMJ performance of female Gaelic footballers was explored by Tucker and Reilly [45] with mean values of 44.9 ± 3.2 cm reported. These values are similar to data reported for female Australian football (45.5 ± 3.1 cm) players [44]. It has been demonstrated that long term (> 10 weeks) plyometric training is an effective method to improve vertical performance jump in female athletes [68]. A 12-week plyometric training intervention combined with regular football training improved explosive strength and improvements transferred to soccer specific skills in female athletes [69]. Previous research in female team athletes supports the inclusion of plyometric training into a multi-faceted training program which can decrease knee valgus and increase ground reaction forces and increase hamstring strength [70–72]. It has been shown that combining plyometric or ballistic movement efforts with other resistance training methods (complex training) can lead to enhanced performance as opposed to plyometric training alone in female team athletes [57]. The utilization of a plyometric training stimulus could be beneficial to improve muscular power in elite female Gaelic team sport athletes. Therefore, future research is needed to determine the efficacy of different muscular power training modalities in elite female Gaelic team sports athletes. In conclusion, muscular power characteristics in elite female Gaelic team sport athletes are lower in comparison to their elite female team sports counterparts.

## Injury incidence, risk and prevention

To embed successful injury management strategies in elite female Gaelic team sports, firstly one must understand the epidemiology of injury incidence and burden. Due to the high velocity, multi-directional, contact nature of Gaelic team sports, female athletes who participate in these sports, especially at an elite level, are inherently at risk of injury [73, 74]. In a self-reported participant injury survey across all levels of camogie, indicated that the most traumatic injury that occurred in the game/training activities was knee ligament damage (21% of all injuries) with over 60.8% of correspondences reporting lower limb injuries [3]. Furthermore, 85% of female correspondents felt obliged to continue playing whilst injured, reporting that they did not want to let their teammates down or believed they could manage the injury themselves [3]. Similarly, it was reported that lower limb injuries were the most frequent in elite camogie (71.4%), with 23.8% of injuries occurring to thigh, knee (19%) and ankle (9.5%) [75]. This study reported that 19% of injuries were a result of sprinting, 14.3% resulting from a change of direction, 9.5% occurring from overloading and 4.8% occurring from a push off [75]. Although there is currently no data on injury rates in elite female Gaelic football, lower limb injuries were the most prevalent (67.09%) and caused the greatest injury burden (276.17 days absent per 1000 h) at a collegiate, sub-elite level [76]. Additionally, hamstring injuries were the most common (21.52%) followed by knee (12.66%), quadriceps (11.39%) and ankle (10.31%). It was reported that knee injuries cause the greatest injury burden (106.46 days absent per 1000 h) [76].

It has been reported that female team sports athletes are 3–6 times more likely to obtain an anterior cruciate ligament (ACL) injury than their male counterparts [77]. This high incidence has been largely attributed to anatomical and hormonal aspects including joint laxity, limb alignment, intercondylar notch proportions, ligament size and hormonal fluctuations [78]. Although there is no existing research on ACL injury rates in elite female Gaelic team sports athletes it is imperative that practitioners use different training modalities (resistance training, plyometrics, development of athletic motor skill competencies etc.) to support structural components and improve neuromuscular control of the knee joint and reduce the incidence of non-contact ACL injuries in field-based sports [54, 79]. It is reported during side cutting maneuvers in which internal tibia rotation occurs in < 50 ms after initial contact in conjunction with reduced semitendinosus pre-activation, is associated with an increased risk of ACL injury [80]. Therefore, it may be worthwhile for elite Gaelic practitioners to consider specific training modalities to replicate forces produced < 100 ms such as strength-speed, speed strength and velocity-based modalities [1, 2].

Developing stronger, resilient elite female Gaelic team sports athletes will enable them to master complex, athletic movements innate to the games and cope with the increased demands of training and competition without injury [2, 81]. An INT approach, which combines strength training, plyometrics and movement skill competencies to maximize female athletic performance [54, 55] has been recommended. This multifaceted training system can assist in the strengthening of tendons, ligaments and bones, enhanced balance and coordination between agonist and antagonist muscle groups and an improved capability to withstand training load from specific sports training and competition [53, 54]. It has been demonstrated that integrative neuromuscular training (INT) designed for female soccer players injury prevention simultaneously improved lower body performance measures and improved movement mechanics [54, 55]. This accentuates the importance of a systematic IPP to decrease the severity of injuries [82]. An applied, minimally invasive example of an INT is the Gaelic specific injury prevention program (IPP) (GAA 15) [83–85]. Both LGFA and the Camogie Association have introduced the GAA specific GAA 15 IPP [84, 85]. The implementation of the GAA 15 IPP led to a 66% (*p* = 0.001) reduction in training injuries, hamstring injuries, and non-contact injuries in a collegiate Gaelic team sports specific IPP intervention group [83]. A survey conducted on Camogie coaches and players reported that only 34% of coaches and 11.8% of players were using the GAA 15 and the Camogie Association should include a mandatory Camogie specific IPP concurrently with coach education programs [85]. The development of enhanced performance characteristics in conjunction with greater compliance of IPP’s could mitigate against the injury risks associated with both elite female Gaelic team sports.

## Anthropometric characteristics

The anthropometric characteristics of athletes are an important health and performance variable [86]. The information is beneficial for coaches and practitioners to inform training practices and nutritional strategies as well as potentially guide playing positions. Currently, there is a paucity of anthropometric data on elite female Gaelic footballers compared to camogie players, as demonstrated in Table 1. Players ranged between 18–29 years of age, 1.58–1.74 m in stature, 56.4–75.9 kg in body mass and had a body fat percentage of 21–26%. The age, stature and mass range of female Gaelic footballers are comparable to other female team sports such as rugby, handball, and hockey [86–88], however, the range observed in female soccer players is far greater and this highlights the heterogeneity among top level soccer players [14]. When comparing the data from earlier studies [43] to the most recent [24, 25, 29, 89] players’ stature and body mass have remained similar despite the sport evolving in recent years and the advent of strength and conditioning programs. Positional differences of female Gaelic footballers have been assessed in one study however, this was carried out on sub-elite players [90]. The authors reported similarities between positions for both body mass and body mass index (BMI), but a ~ 3–7 cm mean difference in stature was observed between midfielders and other positions. However, it is unclear if these positional differences exist at an elite level.

**Table 1:** The anthropometric characteristics of elite female Gaelic team sport players

Reference	Country	Sport	Standard	Age (years)	Stature (m)	Body mass (kg)	% Body fat
O’Grady et al. [29] *n* = 43	Ireland	Camogie	Elite	23 ± 5.0	1.74 ± 0.05	68 ± 9	–
Connors et al. [47] *n* = 45	Ireland	Camogie	Elite	23.3 ± 3.47	1.68 ± 0.06	68.4 ± 7.4	–
Renard et al. [89] *n* = 328	Ireland	Camogie, GF		23.7 ± 3.0	1.66 ± 0.08	65.8 ± 9.1	–
Young et al. [24] *n* = 36	Ireland	Camogie	Elite	23 ± 4.0	1.68 ± 0.05	65 ± 6.0	–
Connors et al. [25] *n* = 24	Ireland	Camogie	Elite	23.5 ± 3.37	1.68 ± 0.06	68.5 ± 7.4	–
Buckley and Blake [75] *n* = *62*	Ireland	Camogie	Elite	22.9 ± 3.6	1.68 ± 0.06	64.8 ± 6.3	–
Keane et al. [43] *n* = *46*	Ireland	GF	Elite	18–29	1.66 ± 0.05	63.7 ± 7.3	23.3 ± 2.3

To date, only one study [43] has reported body fat percentage values for elite female Gaelic footballers using a 4-site skinfold assessment method. The body fat percentage values reported by Keane et al. [43] were less than the non-exercising controls in the study but are also comparable to elite female handball [87] and hockey players [88] In contrast, female soccer players tend to have a lower body fat percentage with ranges of 15–22% reported [14] whilst elite rugby league players have slightly higher values typically ranging from 23–39% depending on playing position [91]. Body composition data pertaining to female GAA players requires updating before positional differences can be explored. Although not Gaelic team sports specific, there are best practice recommendations in terms of assessing body composition although this will be dependent on what equipment is available. Where feasible, DEXA or skinfolds (ISAK accredited) should be used [92].

## Nutritional considerations

Nutrition plays a significant role in athletic performance, achieved through the dietary influence on body composition, training adaptation, and recovery [93, 94]. There has been growing interest in the nutritional requirements of Gaelic team sport athletes and subsequently, a number of recent nutritional studies have been conducted. However, to date many of these studies and subsequent recommendations are based on male athletes and as such there is an absence of sex specific nutrition guidelines. This is a vital first step in promoting the health and performance of elite female Gaelic team sport athletes.

### Energy and macronutrient intake

Research has consistently shown sub-optimal dietary practices amongst elite and sub-elite male Gaelic team sport athletes, most notably failure to meet both energy and carbohydrate requirements. Gaelic football players dietary intake has been identified as inadequate to meet recommendations, with average energy deficits of 12.3% per day [95], carbohydrate intakes of 3.4–3.7 g kg day^−1^ [95–97] lower than minimum recommendations of 5 g kg day^−1^. It remains to be established if similar dietary intakes are adopted by female Gaelic team sport athletes, however similar findings have been reported with other female field-based team sport athletes [98–100]. Not only does an inadequacy of energy and carbohydrate intake in a team sport context limit substrate availability during training and matches as well as impair recovery [101], if athletes have inadequate energy intake to match exercise energy expenditure, they are described as having low energy availability (LEA) [102] or relative energy deficiency in sport (RED-S) [103]. Growing evidence indicates that unhealthy dietary behaviors and negative body image issues are common in both male and female athletes, but it is still widely accepted that female athletes are more likely to experience LEA. The underlying cause of RED-S is LEA and is calculated by subtracting exercise energy expenditure (EEE), kcal/d from tkcal/d. If the RED-S syndrome is present in an athlete, either inadvertently or through purposeful dieting or disordered eating, athletes can experience increased fatigue, injuries or illness, nutrient deficiencies, menstrual dysfunction, poor bone health, and lack of improvement in performance. In addition, athletes can experience impairments in metabolic rate, immunity, protein synthesis and cardiovascular health [103]. To date, there is limited research into the prevalence of LEA in female Gaelic team sport athletes' players. One study examining the prevalence of LEA in athletic and recreationally active females in Ireland, including 118 Gaelic footballers and 45 camogie players, found 40% (39.7%, n = 331) of participants were classified as being ‘at risk’ of LEA according to the Low Energy Availability for Females Questionnaire scoring system [104]. Furthermore, risk was 1.7 and 1.8 times more likely to occur among elite (intercounty) athletes compared to those who were recreationally active. Further research on energy availability in elite female Gaelic team sports is needed to consolidate these findings.

On the contrary, elite and sub-elite male Gaelic team sport athletes have been shown to exceed protein and fat recommendations with protein intakes of 1.9–2.1 g kg day^−1^ [96, 97, 105] towards the higher end of recommendations and fat intakes of 31–37.5% of total daily energy intake (TDEI) also at the higher end of recommendations [106]. Over consumption of protein and fat has previously been associated with reduced carbohydrate intake in elite male soccer players [107] and may partly account for the inadequate carbohydrate intake observed in male Gaelic team sport athletes. On the contrary, it has been suggested the female athletes are more likely to be at risk for low protein intakes [108]. Currently it is not known if this high fat and high or low protein eating behaviors are seen in female Gaelic team sport athletes and this warrants further investigation.

### Micronutrient intake

With regards to micronutrient intake, active females often report low levels of micronutrients especially in cases where energy intake is being restricted, food groups are eliminated, a special diet is followed (e.g., vegan, vegetarian, or fad diet), the athlete has an eating disorder, or there are health issues that alter nutrient absorption [108–110]. Inadequate intakes of calcium and iron are common in females, with inadequate iron intakes previously documented in female team sports [111]. In Ireland, vitamin D insufficiency and deficiency is pervasive in athletes, and previous studies have identified a particularly high prevalence of vitamin D insufficiency/deficiency in Gaelic footballers [112, 113]. To date, there has only been one study (abstract only) that has investigated the dietary intakes of calcium, iron, and vitamin D in sub-elite (n = 23) and elite (n = 26) female Gaelic footballers [114]. Overall, median intakes of calcium, iron and vitamin D in both sub-elite and elite footballers were lower than the respective recommended daily intake (RDA) [114]. In addition, reported iron intakes of sub-elite players were significantly lower than elite players (*p* < 0.05), with no differences in calcium and vitamin D intakes between sub-groups. Interestingly, a minority of elite players (n = 2) in this study met the RDAs for calcium, iron, and vitamin D with the aid of dietary supplements [114]. A recent addition to the literature investigating the sex differences in prevalence of dietary supplement use in elite athletes, participating in individual and team sports reported that supplement use was slightly higher in males than in females however female athletes appeared to consume more iron supplements and supplements intended to improve health [115]. To date, there has been no research into supplement usage amongst female or male Gaelic team sport athletes.

### Hydration

The detrimental effects of dehydration on performance have been well documented including, an increase in core body temperature, cardiovascular strain and glycogen utilization and impaired cognitive performance [116, 117]. Despite the obvious benefit of starting exercise in a euhydrated state, many athletes begin exercise already dehydrated [118]. There is a dearth of literature on the hydration status of athletes in Ireland; the majority having focused on men’s Gaelic football [119, 120]. However, one study that included sub-elite female Gaelic team sport athletes found that most participants were euhydrated before and after exercise [112]. This study reported that 25% of the cohort were dehydrated pre and post exercise which is lower than their male counterparts (35 and 48% respectively). This may be in part explained by the lower sweat rates in female athletes [116] or the reliance on percentage body weight loss as criterion for dehydration post-exercise [120]. In contrast, an earlier study by Newell and colleagues reported only 10% of elite Gaelic football players (all males) commenced exercise in a dehydrated state [120]. Further research with elite male and female Gaelic players using validated measures both pre and post exercise are needed.

### Nutritional knowledge

Nutritional knowledge has been shown to play a significant role in adopting optimal nutrition practices, such as sufficient macro- and micronutrient intake. A previous investigation reported inadequate levels of nutrition knowledge in female Gaelic team sport players (football and camogie). It was reported that their mean nutrition knowledge score was 46.0% ± 11.8% and classified as ‘poor’ [89]. Players scored better on food-based questions yet lacked knowledge surrounding specific macronutrient recommendations. Interestingly, elite players scored greater (+ 4.5–5.9%, *p* < 0.05) than sub elite players. Players with higher levels of general education, history of formal nutrition education and previous advice from a nutritionist also presented greater nutrition knowledge (+ 3.7–7.5%, *p* < 0.05). The poor nutrition knowledge of female Gaelic team sports players may have negative consequences for their dietary behavior. Despite a current lack of dietary investigation within the population, other female team sport athletes with poor nutritional knowledge have been shown have insufficient energy and carbohydrate intake [98, 99]. Similarly, this relationship between poor nutritional knowledge and sub-optimal dietary intake has been seen in male Gaelic football players [95, 97]. As such it would be beneficial to measure dietary intake and assess nutritional knowledge simultaneously in elite female Gaelic team sport athletes to see if knowledge translates to practice. In addition, focus should be placed on strategies to enhance nutrition knowledge of sport-specific concepts, addressing macro and micronutrient recommendations as players scored poorest on these aspects.

## Female physiology

### The menstrual cycle (MC)

The MC results in large variations in both oestrogen and progesterone concentrations [121]. Although there is no research available on elite female Gaelic team athletes, these variations in reproductive hormones may influence injury risk and performance. The effect of estrogen has been suggested as a possible injury mechanism for ACLs in female athletes [122], however there is a lack of agreement regarding the influence of sex hormones on ACL injury rates. It has been reported that female soccer players had an increased incident rate during the luteal phase of the MC [123] Martin et al*.* [124] reported that injury rates were 47% and 32% greater in the late follicular phase in comparison to the early follicular and luteal phase. However, caution is needed in the interpretation of these studies as the methodological process (small sample size), ecological validity and terminology (phase definition and confirmation) may have affected the interpretation of the original results.

As females train and compete during all stages of their MC, the potential impact on performance is of worthy consideration. There has been some research conducted in the area with conflicting findings to date and no consensus on whether performance is affected by MC phases [125, 126]. A recent meta-analysis showed that exercise performance may be trivially reduced during the early follicular phase of the MC, compared to other phases [125]. However, it must be noted that there was large between-study variation and as with most research in this area, participants were studied at a group level. As it is commonly known the effects of the menstrual cycle are highly individualized, it is recommended that a personalized approach should be taken whereby players track their own cycles and any perceived phase-related effects on performance should be noted.

### Hormonal contraceptives

There is a plethora of hormonal contraceptives available including oral contraceptives (OC), implants, injections patches, and intra-uterine systems [127]. OCs appear to be the most popular with Martin et al. reporting that 49.5% of 430 elite female athletes surveyed from 24 different sports were currently using OC and 69.8% had used the OC previously. This study also revealed that female athletes reported the ability to regulate menstruation during competition/training as a positive effect and reason for using OCs [127]. To date, there has been no research conducted on the use of hormonal contraceptives in elite female Gaelic team sports players or the reasons for initiation. Furthermore, there are conflicting reports on the effects of OC use on exercise performance throughout the literature [128, 129], with the most recent meta-analysis reporting that OC use might result in slightly inferior exercise performance when compared to naturally menstruating women, however any affect is most likely trivial [129]. Further research is necessary to determine whether the use of OC has positive, negligible, or detrimental effects on female athletic performance with regards to all sports [14].

### Limitations and future recommendations

**Fig. 1:**
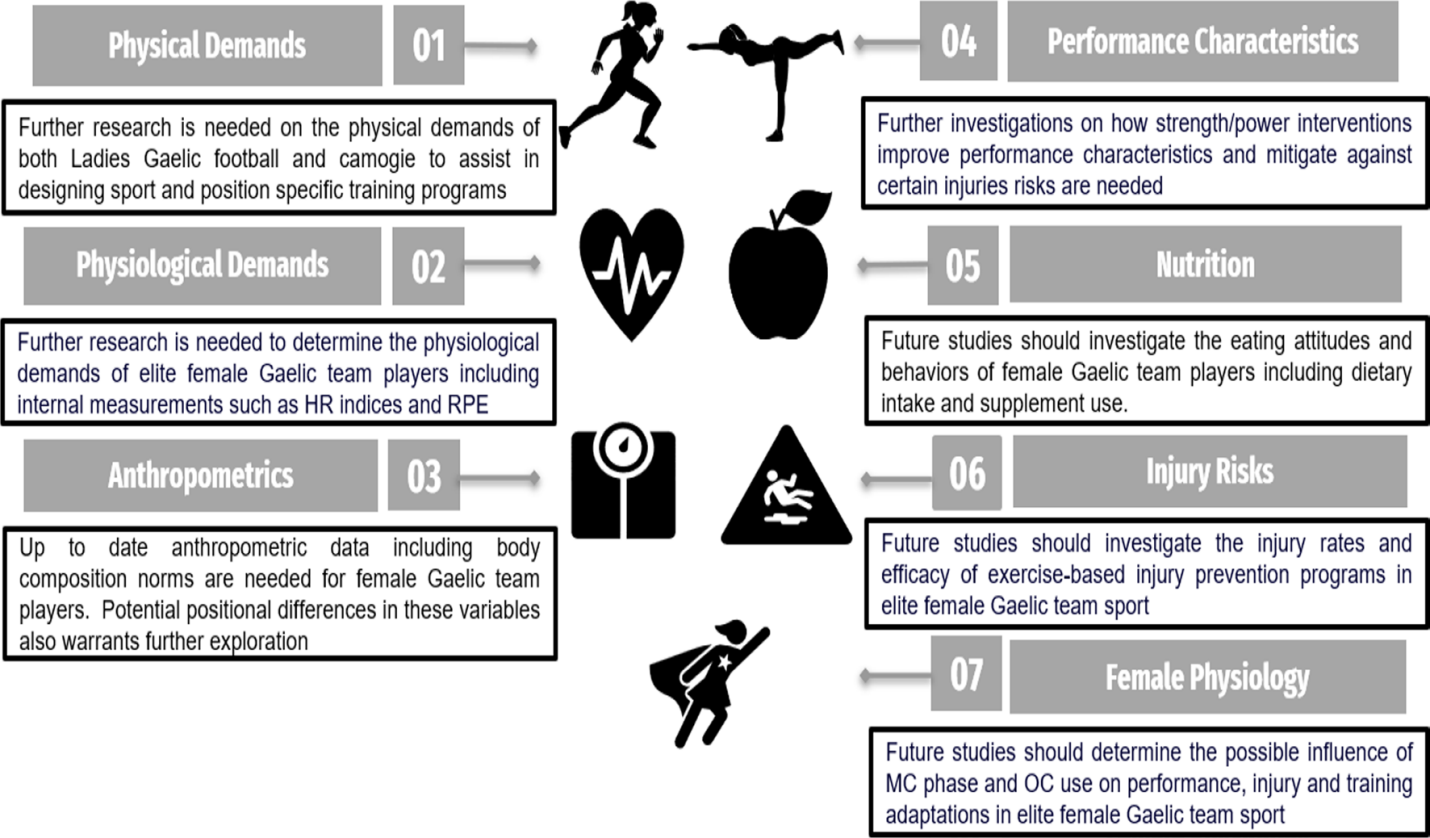
Future research directions in elite female Gaelic team sports

It is important to note the limitations of the current review and in the literature. A narrative style of review was chosen as it was not possible to adopt a systematic approach due to the paucity of research currently available in the field of elite female Gaelic team sport players. In addition, this literature review focused on seven key research topics that are of pertinent interest to researchers and practitioners working in Games, nonetheless, there are other performance parameters that have not been discussed, due to the lack of existing literature. Finally, some of the studies included in the review are of mediocre quality and often include small sample sizes, as such results need to be interpreted with some degree of caution. This review has summarized the available research, as well as highlighting knowledge gaps and areas of future research needs within the domain of elite female Gaelic teams sports. These areas for future research have been highlighted in the individual sections, with a summary provided in Fig. 1.

## Summary and conclusion

Interest in female athletes has grown exponentially over the past decade, with recent research efforts focusing on the match demands, physiological characteristics as well as specific health and performance considerations in female intermittent team-based sports. To the best of the authors knowledge, this is the first review summarizing the current research available in elite female Gaelic team sports, with a focus on seven specific areas including: physical and physiological demands, anthropometric and performance characteristics, injury risk, nutritional considerations, and female physiology.

In summary, there appears to be unique physical demands data in match play across positions in camogie with midfielders having greater total and sprint distance decrements. There is currently no comparative data available in ladies Gaelic football. Similarly, there is no research available on the physiological demands of both elite female Gaelic team sports. According to existing literature, performance characteristics such as speed and power are lower in this population compared to other elite female team sports. Based on the research available, the anthropometric characteristics of elite female Gaelic team sport players appear homogenous with some positional differences in stature observed at a sub-elite level. Previous research has demonstrated a high prevalence of lower limb injuries in female elite Gaelic team sports with 85% of players reporting they felt obliged to continue playing whilst injured. The provision of quality, evidence-based strength & conditioning such as the Gaelic specific IPP could help mitigate these injury risks with positive results already observed at a sub-elite level. With regards to nutrition, female Gaelic team sport players have been shown to have inadequate nutrition knowledge which may put them at a higher risk of LEA. In addition, previous research has shown sub-optimal intakes of certain micronutrients including vitamin D, iron, and calcium in this population. Positively, it would appear female Gaelic team sport players adopt appropriate hydration practices. Finally, although menstrual cycle phase and oral contraceptives have been shown to influence performance in other female intermittent sports, to date there has not been any research carried out in elite female Gaelic team sport players.

To conclude, the current work has summarized the existing literature in the area and created a roadmap for researchers in highlighting the existing knowledge gaps that need to be addressed in this field. Addressing these, would help inform future recommendations and make it easier for practitioners to adopt an evidence-based approach when working with elite female Gaelic team sport players.

## Data Availability

Not applicable.

## Abbreviations

AFL: Australian Rules Football
GPS: Global position system
m: Meters
m min^−^^1^: Meters per minute
km h^−^^1^: Kilometers per hour
HR: Heart rate
HR_max_: Maximum heart rate
HR_peak_: Heart Rate peak
b min^−^^1^: Beats per minute
mL kg^−1^ min^−1^: Milliliters per kilogram per minute
IMTP: Isometric mid-thigh pull
N: Newtons
N/kg: Relative peak force
RFD: Rate of force development
s: Seconds
CMJ: Counter-movement jump
RSI: Reactive strength index
ACL: Anterior cruciate ligament
ms: Milliseconds
INT: Integrated neuromuscular training
IPP: Injury prevention program
GAA: Gaelic Athletic Association
LGFA: Ladies Gaelic Football Association
BMI: Body mass index
DEXA: Dual-energy X-ray absorptiometry
ISAK: International Society for the Advancement of Kinanthropometry
LEA: Low energy availability
REDS: Relative energy deficiency in sport
EEE: Exercise energy expenditure
Kcal/d: Kilocalorie
TDEI: Total daily energy intake
RDA: Recommended daily allowance
MC: Menstrual cycle
OC: Oral contraceptives
RPE: Rate of perceived exertion
